# Age- and Sex-Specific Trends in Lung Cancer Mortality over 62 Years in a Nation with a Low Effort in Cancer Prevention

**DOI:** 10.3390/ijerph13040362

**Published:** 2016-03-25

**Authors:** Ulrich John, Monika Hanke

**Affiliations:** 1 Institute of Social Medicine and Prevention, University Medicine Greifswald, Greifswald D-17489, Germany; hanke@uni-greifswald.de; 2 German Center for Cardiovascular Research (Deutsches Zentrum für Herz-Kreislauf-Forschung e.V.), Berlin D-13347, Germany

**Keywords:** lung cancer mortality, death age, vital statistics, education, female

## Abstract

*Background:* A decrease in lung cancer mortality among females below 50 years of age has been reported for countries with significant tobacco control efforts. The aim of this study was to describe the lung cancer deaths, including the mortality rates and proportions among total deaths, for females and males by age at death in a country with a high smoking prevalence (Germany) over a time period of 62 years. *Methods:* The vital statistics data were analyzed using a joinpoint regression analysis stratified by age and sex. An age-period-cohort analysis was used to estimate the potential effects of sex and school education on mortality. *Results:* After an increase, lung cancer mortality among women aged 35–44 years remained stable from 1989 to 2009 and decreased by 10.8% per year from 2009 to 2013. *Conclusions:* Lung cancer mortality among females aged 35–44 years has decreased. The potential reasons include an increase in the number of never smokers, following significant increases in school education since 1950, particularly among females.

## 1. Introduction

International evidence had revealed a trend towards a leveling out or even a decrease in lung cancer mortality among women aged 30–49 years after the year 2000 [1]. However, from 1985 to 2000, the lung cancer mortality for this age group increased in 15 of 32 countries in the European region [1]. In the United States, lung cancer mortality had already decreased among females since 1985 [1]. Females who were born after 1940 seemed to have lower lung cancer mortality rates than those born from 1930 to 1940 [2]. In the UK, the lung cancer mortality rate of females aged 35 years to 54 years at their time of death was 16 per 100,000 population before 1980 and 12 per 100,000 population in 1997 [3].

The rates of lung cancer deaths of young people may serve to estimate the effects of smoking reduction in different nations. If the effects of preventive interventions are to be detected at an early time point, pragmatic estimators are needed. One approach is to analyze the lung cancer deaths of young people across birth cohorts [4]. It may offer evidence about the changes in the smoking behavior in the population under observation. These are likely to include changes in the proportion of never smokers but not changes of quit rates [4]. Quit rates include the disadvantage that even after more than 30 years of abstaining from smoking, the risks of lung cancer death have been reported to be higher for those who had once smoked than among never smokers [5]. In the United States from 1990 to 1994 and from 1995 to 1999, the lung cancer deaths of individuals aged 30 years to 39 years decreased in states with high prevention efforts and increased in states with low prevention efforts [6]. In California, a state with particularly high prevention activity, the lung cancer deaths among women aged below 75 years decreased from 1990 until the end of the observation period in 2005 [7]. In England, the lung cancer mortality of men and women below 60 years of age decreased from 1950 to 1989 compared to those who were older [4]. The potential reasons include a reduction in the number of smokers and changes in the cigarette components. Another reason for decrease in lung cancer deaths might be due to improvements in school education. The data consistently revealed that higher levels of education are related to lower smoker rates than lower levels of education [8].

We sought to analyze lung cancer mortality in Germany per year, stratified by age and sex. In addition, the proportions of lung cancer deaths among the total deaths are provided to analyze the specificity of the mortality changes for lung cancer. Moreover, the numbers of lung cancer deaths are provided because they may serve as an easy-to-use indicator of public health. All findings refer to the years from 1952 to 2013.

## 2. Materials and Methods

To estimate the number of lung cancer deaths, we used the vital statistics of Germany for the years from 1952 to 2013. The aggregated data included the number of total and cause-specific deaths per calendar year. Cancer of the trachea, bronchus or lungs has been uniformly used from 1952 to 2013. In the years 1952 to 1967, this was equivalent to code 223, cancer of the trachea, bronchus or lungs of ICD-6 and ICD-7; in the years 1968 to 1997, this was equivalent to the same disease group as code 162 in ICD-8 and ICD-9; and since the year 1998, this was equivalent as codes C33 (cancer of the trachea) and C34 (cancers of the bronchus or lungs) in ICD-10 [9]. The age at death was provided in 5-year age groups.

We used the following ages at death groups: 35–44, 45–54, 55–64, 65–74, 75–84, and 85 or older. Additionally, we analyzed the ages 35–39 and 40–44 years to answer the question of whether the potential trends in this young age group might be restricted to those aged 35–39 years. The age limit of 35 years was chosen because lung cancer was rare at ages below 35 years. For the years from 1952 to 2013, there were a maximum of 53 deaths per year, or 0.8% of all deaths among women younger than 35, and a maximum of 78 deaths per year, or 0.4% of all deaths among men at this age. Until 1990, our analysis was based on the data for the Federal Republic of Germany only. Germany was divided into two nations until 1990. After that, the data of the reunited Germany were used, which included the populations of both former states. For school education, we used reports of the Statistical Office of the German Reich and the Federal Statistical Office about the numbers of residents who successfully completed the highest degree in the German school system, which included 12–13 years of schooling and is known as the general university-entrance diploma [10,11,12,13]. The school data were only available for residents who had finished school in 1926 or later. We used these data as estimates for the proportion of those with highest school degree among the female and the male birth cohorts 1901 or younger.

The data analyses were performed by stratifying the information by age and sex for each of the 62 years from 1952 to 2013. For each year, we used three indicators of lung cancer death: the number of lung cancer deaths per 100,000 population of the same 5-year age group and sex (lung cancer mortality), the proportion of lung cancer deaths among the total deaths of the same age and sex (lung cancer death proportion), and the absolute number of lung cancer deaths (number of lung cancer deaths). To calculate the trends, we used the program Joinpoint (Version 4.1.1.3, December 2014; National Cancer Institute, Bethesda, MD, USA) [14,15]. The joinpoint regression analysis provides a number of joinpoints that define segments of time. We describe the time segments as trends. The maximum number of trends was defined to be 5 to allow for sufficient detail in the analysis. We also calculated regression analyses without any joinpoint to test whether a significant trend existed over the entire period of 62 years. Significant trends are interpreted as increases or decreases. Insignificant trends are denoted as stable or leveled. Each of the 62 years under observation was included in the joinpoint regression program. We calculated the Joinpoint analysis for each age group stratified by sex. To provide a more detailed analysis of the birth cohort effects, we performed an age-period-cohort analysis [16] using Stata 14.1 (StataCorp LP, College Station, TX, USA). Our analysis included a general linear model for the prediction of lung cancer with sex and education as covariates [16]. Incidence rate ratios for men compared to women and for percentage point individuals with the highest school education were provided. For education, we used the proportion of males and females who had the highest school degree (12–13 years of school) compared to residents with lower levels of education. Among females, this proportion increased from 0.3 % among all female residents born in 1901 to 23.0% among those born in 1973 (Table 1). Among males, the proportion increased from 3.0% among all male residents born in 1901 to 21.4% among those born in 1973. We replaced the missing values for the percentage of females with the highest education among all females born between 1862 and 1900 with 0.3%, and we replaced the missing values for the percentage of males born between 1862 and 1900 with 3%. 

## 3. Results

**Figure 1:**
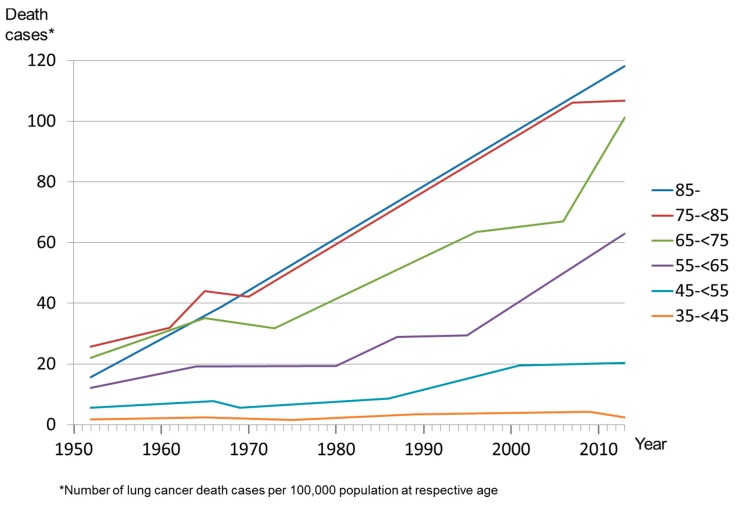
Death cases per year, females.

The lung cancer mortality for women aged 35–44 years increased from 1.7 per 100,000 population (of females) at this age in 1952 to 4.6 in 2005; the number decreased to 2.4 in 2013. The lung cancer death proportion of women aged 35–44 years increased from 0.7% in 1952 to 5.5% in 2005, and then it decreased to 3.3% in 2013. The statistical analysis of the trends revealed that after stabilizing in the years from 1952 to 1965 and after a decrease from 1965 to 1975, lung cancer mortality among women aged 35–44 increased by 6.5% per year from 1975 to 1989 (Table 2; Figure 1). After 1989, lung cancer mortality stabilized and decreased by 10.8% per year from 2009 to 2013. The proportion of lung cancer deaths among total deaths of women aged 35–44 years increased from 1975 to 2009 and decreased from 2009 to 2013. The lung cancer mortality for women aged 35–39 decreased by 2.0 percentage points annually from 1991 to 2013. The number of lung cancer deaths of women aged 35–39 years showed a decrease of 6.2 percentage points in the most recent trend: 1999 to 2013. Among the women aged 45–54 years who died of lung cancer, mortality leveled out from 2001 until 2013. Lung cancer mortality of women aged 55 or older increased until 2013 with the exception of those who died at age 75–84.

**Figure 2:**
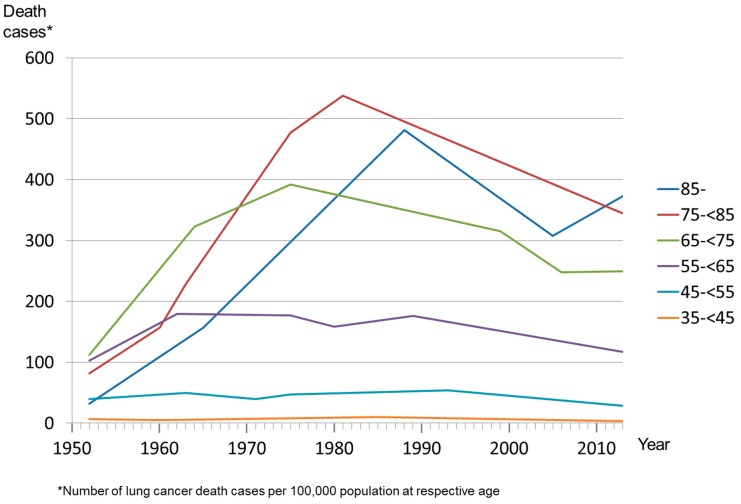
Death cases per year, males.

The lung cancer mortality for men aged 35–44 years increased from 6.7 per 100,000 in 1952 to 10.6 in 1983 and then decreased to 3.7 in 2013. The trend analysis revealed that for men aged 35–64 years, the lung cancer mortality rate decreased from 1952 to 2013 when the entire time span 1952–2013 is considered (Table 2). In detail, lung cancer mortality for men aged 35–44 decreased from 1985 after an increase from 1960 to 1985, and the proportion of lung cancer deaths among total deaths decreased or stabilized since 1988. In lung cancer deaths at age 45 or older, there was stabilization or decrease in the most recent trend of all three indicators of lung cancer death until 2013 (Figure 2). The exceptions were three trends at age 75 or older. The age-period-cohort analysis revealed significant age, period, and cohort effects on lung cancer mortality, both among females and males. The incidence rate ratios for the age, period, and cohort effects were significant at the .001 level. According to the general linear model, the data revealed that men had a six-fold higher rate of lung cancer mortality than women (incidence rate ratio 5.98; 95% confidence interval 5.94–6.02). The lung cancer mortality of residents who had the highest level of education decreased by 0.03 per percentage point individuals with highest education among the age-adjusted general population of same sex (incidence rate ratio 0.97; 95% confidence interval 0.967–0.971).

## 4. Discussion

The main finding of this study is that there was a decrease in the birth cohorts of women who died from lung cancer at a young age in recent years. For women who died at age 35–44 years, the lung cancer mortality increased from 1975 to 1989, leveled out from 1989 to 2009, and started to decrease in 2009. The lung cancer mortality of women aged 35–39 years started to decrease in 1991. This trend lasted until the end of observation period in 2013. The number of lung cancer deaths of women aged 45–54 years showed a somewhat more moderate trend by stabilizing mortality and the proportion of lung cancer deaths among all deaths. The number of lung cancer deaths of women age 55 years or older increased in the most recent trend until 2013. These findings suggest that changes started in the most recent cohorts among those who died at a young age. The decrease in deaths among women from the youngest cohort is suggested by the data on mortality, the proportion of lung cancer deaths among total deaths, and the number of lung cancer death. The lung cancer mortality of men aged 35–44 years had already decreased since 1985. These decreasing trends occurred in a nation that had not undertaken any significant efforts to reduce smoking in the years before the decreases or stabilizations in the numbers of lung cancer deaths had begun.

Several reasons might explain the reduction in the incidence of lung cancer, such as changes in smoking habits, changes in tobacco products, changes in additional risk factors, and improvements in medical treatment. Smoking may have been less attractive among females born after 1950 than among females born before 1950. The trend data on smoking in Germany using an estimation based on taxed tobacco products revealed that after 1952, an increase in smoking rose to 2919 cigarettes or equivalents of other tobacco products per resident aged 15 years or older; however, in 1971, tobacco sales stabilized, and beginning in 2002, the number of tobacco sales had decreased [17]. Decreases in the numbers of lung cancer deaths among adults age 30–39 years have been reported in the United States, particularly for states that take strong action to reduce the number of smokers, such as California [6]. This was not the case for Germany. Two reasons remain for the reduction in the number of lung cancer deaths: improvements in education and increased public awareness of the health hazards of smoking. In Germany, the rate of those with a school education of 12 or more years has steeply increased since 1950. The rate was 3.1% among the female population at age 19 in 1950, 4.3% in 1960, and 23.0% in 1990. Among men, there was a smaller increase from 6.1% in 1950 to 21.4% in 1990 [10]. The findings of the age-period-cohort analysis support the role of education. The data revealed that an inverse relationship exists between the increasing proportion of those with the highest education among the cohorts and decreased lung cancer mortality in both females and males.

In many countries, the evidence showed that the proportion of smokers was lower as the education level increased among the population [18]. In Europe, the data from 1994 to 2004 revealed that among less educated women aged 25–39 years, there were more ever smokers than among those of the same age who were more educated [19]. For female smokers in Europe, the inequalities increased between 1985 and 2000 [20]. Accordingly, lung cancer mortality may be expected to decrease for higher educated women. In France, fewer lung cancer deaths were observed among higher educated females aged 56 years or younger than among those who did not graduate from school [21]. On the contrary, among females aged 60 years or older, the higher educated women included more ever smokers than the lower educated women [22]. This corresponds to the model of the stages of smoking and the lung cancer epidemic [23]. In its early stages, tobacco smoking may be more prevalent among higher educated women than among lower educated women; this trend largely depends on social norms that were in favor of smoking and on insufficient public awareness of its health hazards during that period. In later stages, education may contribute to better recognition of these risks among female cohorts in the higher social classes, which is then followed by increasing rates of never smokers among these women.

Additionally, the public awareness about the health hazards of smoking among the higher educated may have been supported by the attitudes and norms against smoking from other nations. This influence is possible, as activities in other European countries and the United States largely date back to the mid-1960s. Furthermore, a general trend towards health may have occurred.

The second potential reason for the reduction in the number of lung cancer deaths is that changes in cigarette composition may have occurred. Accordingly, the younger cohorts of smokers would have preferred less harmful cigarettes than the older female smokers. This less harmful composition would have caused significant reductions in the numbers of lung cancer deaths. The evidence is not consistent with this assumption [24], although the risk for adenocarcinoma has increased [24], which has been attributed to changes in cigarette composition since the 1950s [24]. Regarding other potential reasons for the reduction in the number of lung cancer deaths, it has been shown that neither exposure to asbestos nor progress in successful lung cancer treatments qualify as reasons for the degree of the reductions [17].

The trend towards decreases in lung cancer deaths may not be explained by prevention efforts in Germany. Germany is one of the European countries with the lowest activity in preventing tobacco-attributable diseases [25]. No significant preventive action had been established in the years before the most recent trends of lung cancer deaths. Legal measures started in 2002, the 2001 retail price per cigarette increased by 51.1% until 2005 [26], taking into account the inflation-adjusted stable cigarette prices for the years 1990 to 2000 [27], as well as presumably before that time.

The approach of analyzing lung cancer deaths in young people has limitations. The number of total deaths is low in younger age groups *cf.* [4]. Second, the data of the former German Democratic Republic could not be considered. The data for causes of death until 1990 were only obtained for the Federal Republic of Germany. Females in the former East German Democratic Republic may have smoked less than their counterparts in West Germany. This is suggested by the lower lung cancer mortality in East Germany compared to West Germany after the German reunification in 1990 [28]. However, the differences in lung cancer mortality between the eastern and the western states of Germany pertain to women aged 50–89 years who died of lung cancer, which is older than the age of death that is in the focus of our analysis. We did not detect any decrease among females aged 50 years and over. Third, the Federal Statistics Office data are the only data source we used. Adequate survey data are missing for smoking in years that are relevant for lung cancer. However, the indicators of lung cancer death are sufficient to draw conclusions about tobacco smoking. Considering the evidence on the tobacco-attributable fraction of lung cancer among females and males, 90% of lung cancer deaths are tobacco-attributable [5,24,29].

## 5. Conclusions

The data analysis revealed that lung cancer mortality among females aged 35–44 years has decreased in recent birth cohorts. Lung cancer mortality may decrease as the rate of residents with higher school education increases.

## Figures and Tables

**Table 1:** Birth year and school education.

Birth Year	Percent with School Education 12–13 Years among 19 Year Olds ^a^	
	Women	Men
1901–1910	0.3	3.0
1911–1920	1.3	5.5
1921–1930	1.2	5.6
1931–1940	3.1	6.1
1941–1950	4.3	7.4
1951–1960	8.2	12.1
1961–1970	15.4	15.6
1971–1973	23.0	21.4

^a^ [10,11,12,13].

**Table 2:** Trends of lung cancer deaths, Germany, years 1952–2013.

Lung Cancer Deaths	Trend Number ^a^										
	1		2		3		4		5		0
	**Years**	**APC ^b^**	**Years**	**APC ^b^**	**Years**	**APC ^b^**	**Years**	**APC ^b^**	**Years**	**APC ^b^**	**1952–2013 APC ^b^**
**Women**											
Age at death 35–44											
Mortality	1952–1965	+1.2 ns	1965–1975	−3.4	1975–1989	+6.5	1989–2009	+0.6 ns	2009–2013	−10.8	+1.63
% of all death cases	1952–1962	+4.3	1962–1975	−1.2 ns	1975–1988	+8.6	1988–2009	+3.2	2009–2013	−9.8	+3.62
Number lung cancer deaths	1952–1965	+2.0	1965–1973	−4.6	1973–2000	+6.0	2000–2009	−2.0 ns	2009–2013	−14.2	+2.64
Age at death 35–39											
Mortality	1952–1964	+2.3 ns	1964–1971	−7.3 ns	1971–1991	+5.3	1991–2013	−2.0			+1.07
% of all death cases	1952–1964	+4.8	1964–1971	−6.5 ns	1971–1987	+8.2	1987–2010	+2.0	2010–2013	−12.7 ns	+3.26
Number lung cancer deaths	1952–1962	+6.0	1962–1971	−6.0	1971–1999	+5.7	1999–2013	−6.2			+2.04
Age at death 40–44											
Mortality	1952–1959	+3.7 ns	1959–1981	−1.0	1981–1986	+15.2	1986–2003	+2.2	2003–2013	−4.6	+1.81
% of all death cases	1952–1960	+4.5	1960–1981	+0.5 ns	1981–1986	+18.2	1986–2009	+3.4	2009–2013	−10.8	+3.69
Number lung cancer deaths	1952–1960	−3.0 ns	1960–1963	+13.4 ns	1963–1975	−3.1	1975–2003	+6.4	2003–2013	−6.8	+2.87
Age at death 45–54											
Mortality	1952–1966	+3.0	1966–1969	−9.9 ns	1969–1986	+2.6	1986–2001	+5.7	2001–2013	+0.2 ns	+2.62
% of all death cases	1952–1966	+3.7	1966–1969	−8.9 ns	1969–1976	+3.3	1976–2004	+6.2	2004–2013	+1.1 ns	+4.21
Number lung cancer deaths	1952–1966	+1.9	1966–1969	−10.6 ns	1969–1988	+3.2	1988–1992	+16.0	1992–2013	+3.8	+3.45
Age at death 55–64											
Mortality	1952–1964	+3.1	1964–1980	+0.6	1980–1987	+6.0	1987–1995	+0.4 ns	1995–2013	+4.6	+2.57
% of all death cases	1952–1966	+4.5	1966–1969	−7.8 ns	1969–1990	+5.2	1990–1993	+0.7 ns	1993–2013	+5.9	+4.22
Number lung cancer deaths	1952–1964	+6.1	1964–1980	−0.9	1980–1983	+11.4 ns	1983–2013	+4.5			+3.32
Age at death 65–74											
Mortality	1952–1965	+3.4	1965–1973	−0.7 ns	1973–1996	+3.2	1996–2006	+0.3 ns	2006–2013	+6.1	+2.27
% of all death cases	1952–1965	+5.4	1965–1969	−3.4 ns	1969–1982	+4.2	1982–1987	+7.7	1987–2013	+4.4	+4.28
Number lung cancer deaths	1952–1965	+6.5	1965–1989	+2.2	1989–1993	+12.8	1993–2002	+1.0 ns	2002–2013	+4.5	+3.64
Age at death 75–84											
Mortality	1952–1961	+3.2	1961–1965	+7.9	1965–1970	−1.2 ns	1970–2007	+2.5	2007–2013	+0.4 ns	+2.45
% of all death cases	1952–1960	+3.8	1960–1965	+9.7	1965–1969	−2.6 ns	1969–2013	+4.4			+4.19
Number lung cancer deaths	1952–1967	+8.0	1967–1973	+2.4 ns	1973–1979	+8.5	1979–2005	+4.2	2005–2013	+1.8	+5.04
Age at death 85 and older											
Mortality	1952–1967	+7.5	1967–2013	+2.3							+3.06
% of all death cases	1952–1981	+5.9	1981–2013	+2.5							+4.08
Number lung cancer deaths	1952–1964	+13.7	1964–1994	+9.1	1994–2013	+3.9					+8.33
**Men**											
Age at death 35–44											
Mortality	1952–1960	–2.2	1960–1985	+2.5	1985–2013	–3.7					–0.51
% of all death cases	1952–1974	+1.3	1974–1988	+4.3	1988–1992	–9.2	1992–2005	+0.5 ns	2005–2013	–2.9	+0.87
Number lung cancer deaths	1952–1960	–3.7	1960–1964	+12.2	1964–1979	+4.0	1979–2003	+0.1 ns	2003–2013	–8.6	+1.02
Age at death 35–39											
Mortality	1952–1987	+1.2	1987–2013	–4.3							–1.00
% of all death cases	1952–1989	+2.1	1989–1992	–9.3 ns	1992–2013	–0.4 ns					+0.55
Number lung cancer deaths	1952–1977	+4.6	1977–1984	–3.9 ns	1984–1999	+2.0	1999–2013	–8.4			+0.43 ns
Age at death 40–44											
Mortality	1952–1986	+1.9	1986–2000	–2.6	2000–2013	–5.6					–0.40
% of all death cases	1952–1976	+1.6	1976–1988	+4.5	1988–1992	–7.6 ns	1992–2005	–0.1 ns	2005–2013	–3.5	+0.90
Number lung cancer deaths	1952–1960	–7.4	1960–1964	+15.7	1964–1980	+4.3	1980–2004	0.0	2004–2013	–8.9	+1.24
Age at death 45–54											
Mortality	1952–1963	+2.3	1963–1971	–3.3	1971–1975	+6.1	1975–1993	+0.5	1993–2013	–3.0	–0.32
% of all death cases	1952–1962	+2.1	1962–1970	–1.4	1970–1989	+2.6	1989–2013	–0.5			+0.93
Number lung cancer deaths	1952–1959	+2.2	1959–1970	–4.2	1970–1974	+10.9	1974–1991	+3.2	1991–2013	–1.3	+1.09
Age at death 55–64											
Mortality	1952–1962	+5.5	1962–1975	–0.1 ns	1975–1980	–2.6	1980–1989	+1.7	1989–2013	–2.0	–0.26
% of all death cases	1952–1962	+4.4	1962–1973	–0.4 ns	1973–1989	+2.2	1989–2013	+0.1 ns			+1.14
Number lung cancer deaths	1952–1963	+9.4	1963–1979	–3.4	1979–1995	+5.4	1995–2009	–3.2	2009–2013	+2.9 ns	+1.05
Age at death 65–74											
Mortality	1952–1964	+9.3	1964–1975	+1.8	1975–1999	–0.9	1999–2006	–3.8	2006–2013	+0.1 ns	+0.48
% of all death cases	1952–1964	+8.7	1964–1997	+1.4	1997–2013	+0.2 ns					+1.96
Number lung cancer deaths	1952–1967	+10.4	1967–1976	+2.8	1976–1988	–3.8	1988–1995	+8.8	1995–2013	–0.1 ns	+2.24
Age at death 75–84											
Mortality	1952–1960	+8.5	1960–1963	+12.7	1963–1975	+6.2	1975–1981	+2.4	1981–2013	–1.3	+1.90
% of all death cases	1952–1964	+9.6	1964–1979	+6.2	1979–1995	–0.2 ns	1995–2000	+4.0	2000–2013	+0.3 ns	+3.20
Number lung cancer deaths	1952–1963	+11.0	1963–1981	+7.5	1981–1999	–0.5	1999–2005	+5.8	2005–2013	+1.9	+4.09
Age at death 85 and older											
Mortality	1952–1965	+11.3	1965–1988	+4.8	1988–2005	–2.5	2005–2013	+2.0 ns			+3.05
% of all death cases	1952–1962	+10.9	1962–1985	+7.1	1985–2004	–0.7 ns	2004–2013	+2.4			+4.07
Number lung cancer deaths	1952–1964	+16.6	1964–1994	+7.8	1994–2004	–2.6	2004–2013	+7.1			+6.81

^a^ Number of trends: 5 or less; all trends were significant at p<.05; ns: not significant. For mortality in 2013, the deaths per 100,000 population in 2012 were used; ^b^ APC: Annual percent changes.
